# Black holes as critical point of quantum phase transition

**DOI:** 10.1140/epjc/s10052-014-2752-3

**Published:** 2014-02-12

**Authors:** Gia Dvali, Cesar Gomez

**Affiliations:** 1 Department für Physik, Ludwig-Maximilians-Universität München, Arnold Sommerfeld Center for Theoretical Physics, Theresienstr. 37, 80333 München, Germany; 2 Max-Planck-Institut für Physik, Föhringer Ring 6, 80805 München, Germany; 3 Theory Department, CERN, 1211 Geneva 23, Switzerland; 4 Department of Physics, Center for Cosmology and Particle Physics, New York University, 4 Washington Place, New York, NY 10003 USA; 5 Instituto de Física Teórica UAM-CSIC, C-XVI, Universidad Autónoma de Madrid, Cantoblanco, 28049 Madrid, Spain

## Abstract

We reformulate the quantum black hole portrait in the language of modern condensed matter physics. We show that black holes can be understood as a graviton Bose–Einstein condensate at the critical point of a quantum phase transition, identical to what has been observed in systems of cold atoms. The Bogoliubov modes that become degenerate and nearly gapless at this point are the holographic quantum degrees of freedom responsible for the black hole entropy and the information storage. They have no (semi)classical counterparts and become inaccessible in this limit. These findings indicate a deep connection between the seemingly remote systems and suggest a new quantum foundation of holography. They also open an intriguing possibility of simulating black hole information processing in table-top labs.

## Introduction

The exchange of ideas between condensed matter and particle physics has a long history. In the present paper we would like to establish one more link: A fundamental connection between the physics of black holes and critical phenomena in quantum Bose–Einstein condensates (BEC) that are formed in ordinary quantum systems such as cold atoms and photon gases.

This connection originates from a recently formulated quantum theory of black holes, according to which black holes represent quantum BEC of gravitons [1–3].

In the usual treatment gravitational systems, such as the black holes (or even an entire Universe), are introduced through the background geometry that they produce. Thus, in the semi-classical approximation, one studies small fluctuations about the background, but the background geometry itself is treated as an intrinsically classical entity. However, in nature there are no truly classical objects, Planck’s constant is non-zero, $\hbar \, \ne \, 0$. So in the semi-classical treatment we are working in the limit in which the quantum constituents of the geometric background cannot be resolved.

Hence, what are the true quantum constituents of the classical geometry?

Just in the same way as a laser beam is an emergent classical description of a large occupation number of photons, the classical geometry must be handled as an effective description of a quantum state with large graviton occupation number. When such a state is a ground state, the gravitational field is effectively a BEC. This is the case for black holes [1–3].

Unlike the photons (which are electrically neutral) gravitons gravitate and can form a self-sustained Bose-condensate, a black hole. The special property of such a condensates is that they are at the point of *maximal packing*. The maximal packing means that the size of the condensate, $L$, depends on the occupation number $N$ in such a way that it is impossible to further increase $N$ without increasing $L$. Essentially, at the point of maximal packing $N$ becomes the sole characteristic of the system. In particular, the size scales as $L\, = \, \sqrt{N} \, L_P$, whereas the coupling between individual particles scales as $\alpha \, = \, 1/N$. Putting it simply, a black hole represents a large-$N$ system in the ’t Hooft’s sense [4], with the critical value of the coupling, $\alpha N \, = \, 1$.

This picture naturally explains all the semi-classical mysteries of black holes. Particularly, the Hawking radiation [5] and the negative specific heat result from quantum depletion of the condensate. The spectrum of the radiation is thermal up to $1/N$-corrections with effective temperature $T\, = \, \hbar /\sqrt{N} L_P$. The resulting heat capacity is obviously negative, since $N$ decreases as a result of the depletion.

It is interesting that emergence of thermality has nothing to do with the temperature of the condensate, but it instead results from the self-similarity of the depletion and leakage, which does not change the $N$-dependence of the black hole characteristics.

Another black hole mystery is the origin of Bekenstein entropy [6] and the quantum mechanism of information storage and processing by a black hole. The scaling of Bekenstein entropy as the horizon area, $S \, \sim \, L^2/L_P^2$, creates the impression that the horizon is a union of $N$ Planck-size pixels each housing a *distinguishable* degree of freedom that can be in a discrete number of degenerate states (e.g., $\pm$) resulting in an exponentially large number of micro-states. Due to this property, these hypothetical degrees of freedom are often called *holographic* [7, 8].

In the semi-classical picture the microscopic origin of these degrees of freedom is mysterious (and as we shall show, inaccessible in principle). Instead in our quantum picture, these degrees of freedom naturally originate as collective quantum excitations of the graviton Bose-condensate, huddled within a mass gap of order $1/N$. In [1–3] we refer to them as *flavors*. These flavor degrees of freedom are intrinsically quantum and they decouple as $1/N$ in the classical limit, as they should. Correspondingly in the (semi)classical limit it becomes infinitely hard to resolve them. This explains why in this limit black holes can store an arbitrary amount of information, without ever releasing it.

If black holes are BEC, then it is natural to expect that at least some of its properties must have counterparts in ordinary BEC [9] such as in systems of cold atoms [10–12] or photons [13]. The purpose of this paper is to establish this connection. The motivation for such an analysis is pretty clear. First, it is of fundamental importance to establish unity of physical phenomena in seemingly remote systems. Secondly, such a connection can potentially enable us to simulate quantum black hole physics in the table-top labs. A potential byproduct of such a simulation can be the use of black hole information storage and processing in cold atomic or photonic systems.

In this paper we shall try to make a first step in this direction and to show that the connection is much deeper than what one would have naively thought.

To summarize our findings briefly:

### Black holes represent Bose–Einstein-condensates of gravitons at the critical point of a quantum phase transition

Quantum phase transitions are well known in condensed matter physics (see, e.g., [14]). The category of quantum transitions that is our main focus was studied recently in cold atomic systems [10–12]. As we shall explain, such phase transitions capture the key physics of the black hole quantum portrait.

The essence of the connecting phenomenon is the following. In the presence of an attractive interaction a fixed size BEC undergoes a phase transition above critical values of the occupation number $N$. The uniform BEC becomes unstable and moves into the phase of a bright soliton. At the critical point Bogoliubov modes become almost degenerate with the ground state, within the energy gap that collapses as $1/N$. These gapless modes reflect the underlying breaking of symmetry and the corresponding appearance of Goldstone modes. At the same point the quantum depletion of the condensate becomes important.

Detailed comparison of the above system with the black hole picture of [1–3] reveals that all the above phenomena have exact counterparts there. Namely, the critical value of the occupation number in the black hole case corresponds to the point of maximal packing (the critical value of the ’t Hooft’s coupling) or equivalently to self-sustainability of the graviton condensate. The gapless Bogoliubov modes are playing the role of the holographic flavors that account for the black hole entropy and the quantum depletion is the Hawking radiation. A brief summary of the black hole—BEC correspondence is

- Maximal packing (self-sustainability) $\, \leftarrow \rightarrow \,$ critical point of a quantum phase transition;
- Holographic degrees of freedom (flavors) $\, \leftarrow \rightarrow \,$ gapless Bogoliubov modes at the critical point;
- Bekenstein entropy $\, \leftarrow \rightarrow \,$ quantum degeneracy of the BEC state at the critical point;
- Hawking radiation $\, \leftarrow \rightarrow \,$ quantum depletion and leakage of the BEC.

It is important to stress that we are not dealing with a crude analogy, but with identical physics. Of course, black holes have some peculiar characteristics (so far) not exhibited by ordinary lab condensates. In the lab systems the critical point is achieved by tuning the external factors (e.g., size of the system, number of atoms and interaction strength). Due to this the depletion puts them away from the critical point.

In contrast, black holes are self-tuned and always stay at the critical point due to the self-similarity of the depletion. Emission of a quantum takes a black hole of graviton number $N$ into the one with $N-1$ with all the characteristics depending on $N$ self-similarly. An analogous effect could in principle be achieved in the lab if one externally adjust parameters in order to track the depletion.

As a final remark, we have suggested that the connection between the maximal packing and the holography must not be limited to the black hole case and must be more general. In particular, as a supporting evidence, by extending this connection to an AdS geometry and treating it as a graviton condensate we have observed [1–3] that it also appears at the point of maximal packing. Moreover, the occupation number $N$ of gravitons in AdS exactly reproduces the central charge of the CFT that is independently predicted by AdS/CFT correspondence [15–18]. Is this a simple coincidence?

The results of the present paper suggest that it is not. It originates from the general feature of an overpacked BEC being at the critical point, where Bogoliubov modes become degenerate and the system effectively becomes conformal. We therefore suggest the following.

### The understanding of classical geometries as BECs at the critical point provides a quantum foundation of holography

The maximally packed systems ($\alpha N = 1$), such as black holes or AdS space, are equivalent to BEC’s at the critical point of a quantum phase transition, and as such they are described by (nearly conformal) physics of degenerate Bogoliubov modes. The degree of conformality should be determined by the depletion properties of the condensate.

The paper is organized as follows.

In the next section we briefly review the essentials of the black hole quantum portrait in terms of BEC of gravitons. In Sect. 3 we make contact between this picture and the quantum phase transition in BECs appearing in condensed matter and atomic systems [10–12] and show that they are governed by the same physics. In order to establish this connection we study a prototype theory of BEC that exhibits critical transition and show that these properties closely match the ones of graviton BEC in the quantum picture of a black hole [1–3]. In Sect. 4 we discuss the peculiarity of the graviton BEC that allows it to be stuck at the critical point, even during the quantum collapse and leakage. In Sect. 5 we discuss why maximal packing (equivalently, the critical point) of BEC is crucial for allowing large entropy and information storage. In Sect. 6 we discuss the generalization of our results to other systems with maximal packing and suggest that this provides an universal quantum foundation of holography. Finally, we discuss some potential implications of our results and give an outlook. We shall set the speed of light to 1, but keep $\hbar$ explicit. We shall ignore all the irrelevant numerical factors.

## Black holes as Bose–Einstein condensates

In this section we shall briefly discuss some essentials of the black hole quantum portrait in order to prepare a ground for establishing the connection with condensed matter systems. For a more detailed discussion the reader is referred to the original papers [1–3].

Gravitons are massless spin-2 particles. The strength of graviton–graviton interaction is measured by a dimensionless coupling ‘constant’,

1 $$\begin{aligned} \alpha \, \equiv \, {L_P^2 \over L^2} \, , \end{aligned}$$

where $L$ is a characteristic wavelength of the gravitons participating in the interaction and $L_P$ is the Planck length. In terms of Newton’s gravitational constant, $G_N$, the Planck length is defined as $L_P^2 \, \equiv \, \hbar \, G_N$. The physical meaning of the above coupling can be understood in simple terms as the relativistic generalization of the Newtonian attraction among two gravitons. Notice that the latter force acting among two non-relativistic massive particles of mass $m$ can be written in terms of $\alpha$ as,

2 $$\begin{aligned} V(r)_\mathrm{Newton} \, = \, - \hbar {\alpha \, \over r} \, , \end{aligned}$$

with the same $\alpha$ given by (1), but with the only difference that for a massive particle $L$ has to be understood as its Compton wavelength, $L \, \equiv \, {\hbar \over m}$. The difference for gravitons is that, because they are massless, the role of the Compton wavelength is replaced by an actual wavelength.

From (1) it is obvious that if wavelengths are large, the interaction among the gravitons is extremely weak. For example, for gravitons of wavelength $L \, = \, 1$cm, the quantum interaction strength is $\alpha \, = \, 10^{-66}$! One would say that for all practical purposes such gravitons should behave as free. However, gravitons are bosons, and because of this their occupations numbers can be very high. In such a case the collective effects become extremely important. The key point of our theory is that gravitons can *self-condense* and these condensates are black holes. As we shall see, because of the nature of the coupling (1) these condensates are very special as they are always at the critical point.

In order to see this let us imagine that we wish to localize as many soft gravitons as possible within a space region of size $L$. In other words we are trying to form a BEC of gravitons of characteristic wavelength $L$ by gradually increasing the occupation number $N$. At the beginning, when $N$ is small, graviton interactions are negligible and we need external sources to maintain the condensate. So for small occupation numbers, the behavior is similar to a photon condensate, which requires external binding potentials. However, as we increase $N$ the effects of the interaction become dramatic. Individual gravitons feel a stronger and stronger collective binding potential and for the critical occupation number,

3 $$\begin{aligned} N \, = \, N_c \, =\, {1 \over \alpha } \, , \end{aligned}$$

the graviton condensate becomes *self-sustained*. This self-sustainability condition can be obtained by equating the kinetic energies of individual gravitons, $E_k \, = \, \hbar /L$, with the collective binding potential, $V\, = \, - \alpha \, N \, {\hbar \over L}$,

4 $$\begin{aligned} E_k \, + \, V \, = \, \left( 1 \, - \alpha \, N \right) \, {\hbar \over L} \, = \, 0 , \end{aligned}$$

which is satisfied for the critical value of $N$ given by (3).

An extremely important property of the critical point is that it also corresponds to the point of *maximal packing*. The concept of maximal packing is that the system is so densely packed that its defining characteristics becomes $N$. In particular,

5 $$\begin{aligned} L \, = \, \sqrt{N} L_P , ~~~ \, \alpha \, = \, 1/N . \end{aligned}$$

But for gravitons at the overpacked point it also means that further increase of $N$ without increasing $L$ becomes impossible. Any further increase of $N$ results in an increase of the wavelength in such a way that the system stays at the maximal-packing point (5).

Notice that (4) clearly indicates that the critical point (5) can be achieved for arbitrary $N$, but it is not enough to see why $L$ cannot decrease beyond $L\, < \, \sqrt{N}L_P$. Naively, such a decrease of $L$ would results into an even stronger bounded system.

Such a collapse of $L$ indeed takes place, but remarkably it cannot take the system out of the critical point (5). The reason is that the decrease of $L$ is balanced by the decrease of $N$ due to quantum depletion and leakage of the condensate. As a result the condensate slowly collapses, but it loses gravitons at the same rate. In this way, the system always stays at the critical point (5).

The reason for the diminishing of $N$ is that the graviton condensate undergoes a quantum depletion and the depleted quanta leak out. The key of this phenomenon is that due to the interaction with their fellow gravitons, some of the bosons get excited above the ground state. But, since the ground-state energy is within $1/N$ from the free-escape point, the excited gravitons that gain energies above this tiny gap leave the condensate and join the continuum. In other words, the condensate starts to leak, with a depletion rate which is essentially given by

6 $$\begin{aligned} \Gamma _{leakage} \, = \, {1 \over \sqrt{N} L_P} \, + \, L_P^{-1} \, {\mathcal {O}} (N^{-3/2}) . \end{aligned}$$

This rate can easily be understood from the following estimate. Since the graviton–graviton coupling in the condensate is $1/N$, the probability for any pair of gravitons to scatter is suppressed by $1/N^2$, however, this suppression is compensated by a combinatoric factor $\sim \, N^2$ counting the number of available graviton pairs. As a result, the rate of the graviton emission from the condensate is simply given by the characteristic energy of the process (inverse wavelength of gravitons).

The above quantum depletion translates into the following leakage law:

7 $$\begin{aligned} {\dot{N}} \, = - {1 \over \sqrt{N} L_P} \, + \, L_P^{-1} \, {\mathcal O} (N^{-3/2}), \end{aligned}$$

where dot stands for the time-derivative.

It is precisely this quantum leakage of the graviton BEC that (only!) in the semi-classical limit becomes Hawking radiation.

The correct understanding of the semi-classical limit is the key for understanding why all the above quantum physics of graviton BEC was missed in the previous analysis.

The semi-classical limit is defined by the following double scaling limit,

8 $$\begin{aligned}&N \, \rightarrow \, \infty , ~~~L_P\, \rightarrow \, 0, ~~~ L\equiv \sqrt{N}L_P \, =\mathrm{finite}, \hbar \, = \, \mathrm{finite}.\nonumber \\ \end{aligned}$$

Thus, in the language of BEC the semi-classical limit is the limit in which all the quantum physics of the condensate decouples as $1/N \, \rightarrow \, 0$ and becomes impossible to resolve. What was a quantum condensate now becomes a collection of an infinite number of infinitely soft non-interacting bosons all the individual identities of which are lost. All the semi-classical black hole mysteries are a direct consequence of this unphysical treatment. One of the consequences is the exact thermality of Hawking radiation.

This immediately follows from the leakage law. In this limit (by rewriting $N$ in terms of the black hole mass) this becomes the Stefan–Boltzmann law for a black hole with Hawking temperature given by $T \, = \, {\hbar \over L}$,

9 $$\begin{aligned} {\dot{M}} \, = - {\hbar \over L^2} . \end{aligned}$$

Notice that the exponential suppression of higher frequencies, usually attributed to the thermality of the source, follows from the combinatorics of the quantum depletion. For example, emission of a graviton with much shorter wavelength, $\sim k^{-1} \sqrt{N}L_P$ (where $k \, \gg \, 1$ is a parameter ) requires a re-scattering cascade process of at least $k$ gravitons in the condensate. Due to the variation of the effective graviton coupling along the cascade the corresponding rate for large $k$ is suppressed by the factor,

10 $$\begin{aligned} \Gamma _{k >> 1} \, \propto \, N^{-k} \, k! \, , \end{aligned}$$

where the extra $k!$ comes from the correction to the graviton coupling for a cascade taking place in $k$ consecutive steps. When $k$ is a fraction of $N$, the suppression factor goes like $e^{-k(1\, + \, ln N/k)}$. In the semi-classical limit (8) the above suppression reproduces the exponential Boltzmann-type suppression, which is typical of the thermal spectrum. Nonetheless the underlying quantum physics of this thermal-like spectrum has nothing to do with the thermality of the source, since condensate is in fact cold, but with the underlying quantum physics of BEC being at the overpacked critical point. We will elaborate further this discussion in the next section after introducing a concrete microscopic model of the Bose condensate.

## Black hole as BEC at quantum phase transition

We now wish to establish the connection between the above-discussed black hole quantum portrait and the critical phenomena in ordinary BEC, such as were observed in cold atoms in [10–12]. However, since we would like to keep our discussion as general as possible, we shall consider a simple prototype model that captures the key features of the phenomenon. Let $\Psi (x)$ be a field operator describing the order parameter of a Bose-gas. The particle number density is given by the correlator $n(x) \, = \, \langle \Psi (x) \Psi (x) \rangle$. The simplest hamiltonian that takes into the account the self-interaction of the order parameter can be written in the form

11 $$\begin{aligned} H\,&= \, -\, \hbar L_0 \int \, \mathrm{d}^3x \Psi (x) \varvec{\nabla }^2 \Psi (x) \nonumber \\&\, - \, g \, \int \, \mathrm{d}^3 x \, \Psi (x)^+\Psi (x)^+\Psi (x)\Psi (x) , \end{aligned}$$

where $L_0$ is a parameter of length-dimensionality, and $g$ is an interaction coupling constant of dimensionality [length]$^3\times$[mass]. Since we are looking for a connection with gravity we assume the interaction to be attractive. We shall put the system in a finite box of size $R$ with periodic boundary conditions $\Psi (0) \, = \, \Psi (2\pi R)$ and with the total particle number being $N$. This implies the normalization condition,

12 $$\begin{aligned} \int \, \mathrm{d}^3 x \, \Psi ^+ \Psi \, = \, N . \end{aligned}$$

Performing a plane-wave expansion, $\Psi \, {=} \, \sum _\mathbf{k} \, {a_\mathbf{k} \over \sqrt{V}} e^{i {\mathbf{kx} \over R}}$, where $V=(2\pi R)^3$ is the volume and $a_\mathbf{k}, a_\mathbf{k}^+$ are particle creation and annihilation operators, $[a_\mathbf{k} a_\mathbf{k'}^+] \, = \, \delta _{\mathbf{k k'}}$, we can rewrite the Hamiltonian as

13 $$\begin{aligned} \mathcal {H} \, = \, \sum _{\mathbf{k}} \, \mathbf{k}^2 a_\mathbf{k}^+a_\mathbf{k} \, - \, {1 \over 4} \, \alpha \, \sum _\mathbf{k} \, a_\mathbf{k + p}^+ \, a_\mathbf{k' -p}^+ \, a_\mathbf{k} \, a_\mathbf{k'} \, , \end{aligned}$$

where $$\alpha \, \equiv \, {4g R^2 \over \hbar V L_0}$$ and $$\mathcal {H}\, \equiv \, {R^2 \over \hbar L_0} \, H$$.

We shall now study the spectrum of low-lying excitations about an uniform BEC. That is, we assume that most of the particles occupy the $k =0$ level and study the small quantum fluctuations about this state. The spectrum of fluctuations is determined by the Bogoliubov–De Gennes equation. In first approximation we can use the Bogoliubov replacement,

14 $$\begin{aligned} a_0^+ \, = \, a_0 \, = \, \sqrt{N_0} \, \simeq \, \sqrt{N} \, , \end{aligned}$$

of the ground state creation annihilation operators into classical c-numbers. Note that this approximation relies on taking $N\, \gg \, 1$ while keeping $\hbar$ different from zero. Keeping only terms up to quadratic order in $a_\mathbf{k}^+, a_\mathbf{k}$ for $\mathbf{k} \, \ne \, 0$, and taking into account the normalization condition (12),

15 $$\begin{aligned} a_0a_0 \, + \, \sum _{{\mathbf {k \ne 0}}} \, a_\mathbf{k}^+ a_\mathbf{k} \, = \, N \, \end{aligned}$$

lead (up to a constant ) to the following Hamiltonian describing the small quantum fluctuations:

16 $$\begin{aligned} {\mathcal {H}} \!=\! \sum _{{\mathbf {k \ne 0}}} ({\mathbf {k}}^{2} \!+\!\alpha N/2) a_{{\mathbf {k}}}^{+} a_{{\mathbf {k}}} \!-\! \frac{1}{4} \alpha N \sum _{{\mathbf {k \ne 0}}} (a_{{\mathbf {k}}}^{+} a_{{\mathbf {-k}}}^{+} \!+\! a_{{\mathbf {k}}} a_{{\mathbf {-k}}}).\nonumber \\ \end{aligned}$$

In order to diagonalize the hamiltonian we perform a Bogoliubov transformation,

17 $$\begin{aligned} a_\mathbf{k} \, = \, u_\mathbf{k} \, b_\mathbf{k} \, + \, v_\mathbf{k}^* b^+_\mathbf{k} \, . \end{aligned}$$

The Bogoliubov coefficients are given by

18 $$\begin{aligned} u,v \, = \, \pm {1 \over 2} \left( {\mathbf{k}^2 \, - \, \alpha N/2 \over \epsilon (\mathbf{k}) } \pm \, 1 \right) \, , \end{aligned}$$

leading to the following spectrum of the Bogoliubov modes:

19 $$\begin{aligned} \epsilon (\mathbf{k}) \, = \, \sqrt{ \mathbf{k}^2 ( \mathbf{k}^2 \, - \, \alpha N)} \, . \end{aligned}$$

The Hamiltonian in terms of $b$-particles is diagonal and has the following form:

20 $$\begin{aligned} \mathcal {H} \, = \, \sum _{\mathbf{k}} \epsilon (\mathbf{k}) \, b_\mathbf{k}^+ b_\mathbf{k} \, + \, \mathrm{constant} . \end{aligned}$$

As is clear from (19) the first Bogoliubov energy vanishes for

21 $$\begin{aligned} N \, = \, N_c = \, {1\over \alpha } \, \end{aligned}$$

and the system undergoes a quantum phase transition. This is exactly the phase transition observed in [10–12]. The essence of this phase transition is that for $N \, > \, N_c$ the first Bogoliubov level becomes tachyonic and the uniform BEC is no longer a ground state. Taking into the account ${1 \over N}$-corrections, it is clear that the gap between the uniform ground state and the Bogoliubov modes collapses to $1/N$ and it becomes extremely cheap to excite these modes. So by quantum fluctuations the system starts to be populated by Bogoliubov modes very easily. This means that the condensate starts to undergo a very efficient quantum depletion. The number density of the depleted $a$-particles to each $\mathbf{k}$-level is given by

22 $$\begin{aligned} n_\mathbf{k} \, = \, |v_\mathbf{k}|^2 \,. \end{aligned}$$

Since $n_\mathbf{k}$ decreases as $1/|\mathbf{k}|^4$ for large $|\mathbf{k}|$, the total number of depleted particles is well approximated by the first-level depletion, which gives

23 $$\begin{aligned} \Delta N \, \sim \, n_1 \, = \, \left( { 1 \, - \, \alpha \, N /2 \over \sqrt{ 1 \, - \, \alpha \, N}} \, - \, 1 \right) \, \simeq \, \sqrt{N} \, . \end{aligned}$$

The striking similarity of the above BEC physics with the black hole quantum portrait suggest that in both cases we are dealing with one and the same physics of the quantum phase transition. Indeed the physics of the graviton condensate is reproduced for the particular case of $L_0 \, = \, R \, = L$ and $g = \hbar \, L_P^2$.

The criticality condition (21) then is nothing but the self-sustainability condition (3) which implies that the graviton condensate is maximally packed (5). The energy gap to the first Bogoliubov level is then given by

24 $$\begin{aligned} \epsilon _1 \, = \, { \hbar \over L \sqrt{N}} \, = \, {1 \over N} {\hbar \over L_P} \, . \end{aligned}$$

This expression summarizes a remarkable property of maximally packed systems, as we shall now see.

### The energy cost of a collective excitation can be made arbitrarily low by increasing the occupation number of bosons in the BEC

Thus, by increasing $N$ one can encode an essentially unlimited amount of information in these modes. Notice that in the semi-classical limit (8) the energy gap collapses to zero and BEC (black hole) becomes an infinite capacitor of information storage!

What we are uncovering is that this is a very general property of overpacked BEC’s which are at the critical point of a quantum phase transition. In both cases, the cold atomic system of [10–12] versus the graviton condensate, the key point is the maximal packing. The overpacking of the system results into the collapse of the mass gap and the Bogoliubov modes become degenerate within a $1/N$-window. These almost-degenerate Bogoliubov modes are the quantum holographic degrees of freedom (flavors) that are responsible both for the entropy as well as for the efficient depletion of the system. Notice that these degenerate Bogoliubov modes are intrinsically quantum and have no classical counterparts. In the classical limit they decouple as $1/N$ and become unobservable.

The way the BEC state acquires entropy at the critical point is easy to figure out. In fact in the homogeneous phase for $N<N_c$ the low-lying states in the Fock space are characterized by $|n,N-n>$ where $n$ represents the number of quanta in the first excited state and $N-n$ the ones in the condensate ground state. The quantum phase transition takes place when $N=N_c$. The specific feature of the quantum phase transition is that all these excited states in the Fock space become quasi-degenerate (at $\frac{1}{ N}$ order) in energy manifesting the underlying spontaneous breaking of symmetry and the appareance of a Goldstone mode. Since at the critical point the number of quasi-degenerate ground states is $O(N)$ and we can effectively define $\sim N$ Bogoliubov quasi-zero modes. In the presence of any additional discrete characteristics of bosons (e.g., such as helicity) the scaling of entropy as $S \, \sim \, N$ is a natural expectation.

In this qualitative approach we do not attempt to get the numerical coefficients. Our target instead is to uncover the quantum physics behind the black hole entropy as the quasi-degenerate nature of the corresponding BEC state at the quantum critical point. In terms of information theory what we observe is that once we reach the quantum critical point we can use the Bogoliubov quasi-zero modes to store information at a minimal cost of energy.^1^

Let us now derive the black hole leakage law (7) from the Bogoliubov treatment of the BEC at the critical point. Equation (23) gives the number of depleted particles in the absence of back reaction. Since we are interested in the time dependence of $N$, we need to divide the number of depleted particles $\Delta N \sim \sqrt{N}$ by the minimal time $\Delta t$ that such depletion takes. This time is given by the time that it takes a number of $\Delta N$ of particles to re-scatter. The time for re-scattering of a single pair is given by

25 $$\begin{aligned} \tau \, \sim \, L \, \alpha ^2 \, N^2 \, \sim \, \sqrt{N}L_P . \end{aligned}$$

Correspondingly, the time for $\sqrt{N}$ such re-scatterings is $\Delta t \, = \, \sqrt{N} \tau \, = \, N L_P$. Thus, the resulting leakage law up to $1/N$ corrections is

26 $$\begin{aligned} {\dot{N}} \, = \, -\, {\Delta N \over \Delta t} = - \, {\sqrt{N} \over N L_P} \, = - { 1 \over \sqrt{N} L_P} \, , \end{aligned}$$

which exactly reproduces (7). Thus, we have reproduced the black hole evaporation law from the depletion of the cold BEC at criticality. At this point it is interesting to observe that the re-scattering time defined above coincides with the causal cell for a speed of *sound* $\alpha ^2 N^2$ equal to the speed of light. This is again a typical property of the quantum critical point that very likely lies at the origin of fast scrambling [19, 20].

Notice that the value we have used for $\Delta N$ was derived in the absence of back reaction. The peculiarity of the graviton condensate allows us to neglect this back reaction. The reason is that the black hole graviton condensate is always at the critical point since $\alpha$ is tracking $1/N$. So the approximation of no-back reaction is always good up to $1/N$. This is why $\dot{N}$ is very well approximated by $\Delta N / \Delta t$. The situation is different for the cold atomic systems, where $\alpha$ is an external parameter and one has to take into the account the back reaction, as it was done in [10]. If $\alpha$ is not tracking $N$, then the change of $N$ by $\Delta N$ offsets $\alpha N$ by $\alpha \Delta N$ and in (23) one has to replace $\alpha N \, \rightarrow \, \alpha N ( 1 \, + \, \Delta N/N)$, which gives $\Delta N \, \sim \, N^{1/3}$. Obviously the black hole quantum phase transition should be characterized by some *critical exponents* roughly characterizing the holographic CFT. However, the Bogoliubov approximation we are considering here is simply equivalent to a mean field approximation.

Before closing this section it would be illustrative to compare the BEC derivation of the depletion with the semi-classical derivation of Hawking radiation in the black hole geometry. In both cases the essence of the derivation lies in the Bogoliubov transformation. In the black hole case and simplifying things representing the near horizon as Rindler geometry, the relevant transformation connects creation–annihilation operators relative to the Minkowski and Rindler vacua. This transformation leads to a typical thermal spectrum $\Delta (N_{\omega }) = (e^{\omega T}-1)^{-1}$ with $T$ the Hawking temperature. In the IR we get $\Delta (N)\sim \frac{T}{\omega }$, while in the UV we get the typical thermal exponential suppression. In the case of the BEC we have derived above the depletion in the IR regime obtaining $\Delta (N) \sim \sqrt{N}$. Nicely enough, this corresponds to the minimal energy $\omega \sim \frac{1}{N}$ in the BEC and to an effective temperature $T \sim \frac{1}{\sqrt{N}}$ in agreement with the depletion law. Moreover the exponential suppression in the UV can be easily understood once we have uncovered the meaning of the black hole entropy. In fact when we consider the emission of a very energetic quanta, we are forced to build up these quanta with a certain number $k$ of soft quanta occupying the ground level of the condensate. This effectively reduces the degeneracy of the BEC ground state by a factor of order $e^k$. In other words when the system emits a hard quantum the price to pay is to reduce accordingly the multiplicity of the quasi-degenerate Bogoliubov modes.

## Being stuck at the critical point

As we said above, the important property of the graviton BEC is the impossibility to enter into the strong coupling regime, $\alpha N \, \gg \, 1$. Despite the fact that black holes deplete and leak gravitons they always remain at the critical point, because leakage is self-similar in $N$.

In order to understand this peculiarity, let us first discuss what is happening in other systems for which entering into the strong coupling regime is possible. For cold atomic BEC’s discussed in [10], the quantum phase transition signals the formation of a *bright soliton*. That is, an overpacked condensate ($N \, \gg \, N_c$) prefers to store particles non-uniformly and store them in higher momentum modes. The critical point (3) marks the transition between the two regimes. The reason we can, in this case, enter into the strong coupling regime is that, although the attractive interaction increases, we can compensate it by the quantum pressure created by higher momentum modes in the band of states that are quasi-degenerate in energy with the uniform BEC ground state. The soliton configuration that represents a ground state in this regime can be well approximated by a localized solution of the Gross–Pitaevskii equation,

27 $$\begin{aligned} i \hbar {\partial \Psi \over \partial t} \, = \, - \, ( \hbar L_0 \, { \mathbf \nabla }^2 \Psi \, + \, 2 g \, \Psi ^+ \, \Psi ) \Psi . \end{aligned}$$

In one space dimension for $\alpha N \, \gg \, 1$ this equation has a well-known exact solution [21, 22], a bright soliton,

28 $$\begin{aligned} \Psi _s(x) \, \propto \, \sqrt{\mu \over g} \, \mathrm{sec}h \left( \sqrt{{\mu \over \hbar L_0}} (x - x_0) \right) , \end{aligned}$$

where $\mu$ is a chemical potential, which from normalization condition scales as $\mu \, \sim \, g^2N^2/\hbar L_0$. Therefore, the argument of the (28) scales $\sim (\alpha N) x/R$.

This system exhibits a Goldstone zero-mode (corresponding to a spontaneous breaking of translational invariance) and higher excitations (breathing modes ) separated by an energy gap.

In three dimensions, however, the solitons are unstable and collapse. This can be understood from the following simple energetics argument. Consider a deformation of the uniform condensate such that we localize most of the particles within a region of size $L$. That is, the order parameter $\Psi$ is localized within the region $L$. Due to the normalization condition (12) the over-density scales as $|\psi |^2 \sim N/L^3$. The energy corresponding to such a configuration from the Hamiltonian (11) is

29 $$\begin{aligned} E \, \sim \, {\hbar L_0} {N \over L^2} \, - \, g {N^2 \over L^3} . \end{aligned}$$

This $L$-dependence has no minimum. It has an extremum at the critical point $\alpha N$ = 1. Beyond the critical point $\alpha N \, > \, 1$ the system collapses towards $L \, \rightarrow \, 0$. The collapse indicates that the system prefers to store more and more quanta into the higher momentum modes and the condensate is getting more and more localized.

What is the connection of this phenomenon to our picture of a black hole? The peculiarity of the black hole graviton condensate is that although it also collapses the collapse takes place through a cascade of successive condensates $N\rightarrow N-1 \rightarrow N-2,\ldots$, all of them at the critical point ! This is due to the fact that the black hole collapses by a quantum depletion which is accompanied by the leakage. Depleted quanta escape and leave the condensate, decreasing $N$ according to (7).

In other words, the quantum-mechanical collapse of the graviton condensate is simply a quantum progenitor of what in semi-classical limit becomes the Hawking radiation^2^.

The fact that collapse of a black hole is happening in a self-similar way, so that the black hole stays at the critical point, can be seen from the energy balance argument similar to (29). For the black hole case ($g \, =\, \hbar L_P^2$ and $L_0 \, = \, L$) this energy balance takes the form

30 $$\begin{aligned} E \, \sim \, \hbar {N \over L} \, - \, \hbar L_P^2 \, {N^2 \over L^3} . \end{aligned}$$

This fixes the critical value $L\, = \, L_P\sqrt{N}$ and the corresponding energy $E_c \, = \, \sqrt{N} {\hbar /L_P}$. Beyond this point the energy balance dictates the system to collapse. But the collapse happens without moving away from the critical point. To see this, let us estimate the energy gained by the system by shrinking its size by

31 $$\begin{aligned} \Delta L \, = - \, L_P^2 /L , \end{aligned}$$

which corresponds to a change of $\Delta N \, = \, - 1$ provided the system stays at the critical point. The corresponding change of energy is

32 $$\begin{aligned} \Delta E \, = \, {\hbar \over \sqrt{N} L_P} \end{aligned}$$

which is exactly the energy needed for a single quantum to leak out and deplete the system self-similarly, as described by equation (7). In fact, as shown in [2], the above quantum collapse effectively can be described in the language of a Landau–Ginsburg Lagrangian for the field $N$,

33 $$\begin{aligned} {\mathcal L}_{LG} \, = \, (\dot{N})^2 \, + \, {1 \over N} L_P^{-2} \, + \, L_P^{-2} {\mathcal O}(1/N^2) . \end{aligned}$$

So the graviton BEC collapses self-similarly due to the fine balance between the depletion and the leakage. Such a balance generically is not exhibited by other collapsing condensates in which the original $N$ particles get redistributed and become localized within a smaller region of space. Such condensates can deplete, but they do not necessarily leak at the same rate. This is because in such systems the deep solitonic phase is usually accompanied by a large escape energy (see, e.g., [10]). In particular, depletion in the solitonic regime is very small and of the same order as depletion in the weak coupling uniform BEC phase.

Notice that the condensate may not be purely gravitational but can also include bosons that are subject to other microscopic forces (e.g., gauge forces) that can counteract the gravitational attraction. In such a case the depletion of the condensate can be suppressed, and collapse can be prevented. Such a condensate, when stabilized at the critical point, describes a quantum portrait of what is classically known as an extremal black hole.

This phenomenon can be described in the language of the following effective Hamiltonian:

34 $$\begin{aligned} H\, = \, \hbar L_0 \int \, \mathrm{d}^3x \, |\varvec{\nabla } \Psi |^2 \, - \, \hbar L_P^2 \int \, \mathrm{d}^3 x \, |\Psi ^+\Psi |^2 \, + \, H' , \nonumber \\ \end{aligned}$$

where

35 $$\begin{aligned} H' \,&\equiv \, \int \mathrm{d}^3x \, {L_P^2 \over \hbar } \, \left| \varvec{\nabla } \int \mathrm{d}^3x' {1 \over | x - x'|} (\hbar L_0 \, | \varvec{\nabla } \Psi |^2 \,\right. \nonumber \\&-\left. \, \hbar L_P^2 \, |\Psi ^+\Psi |^2 )\right| ^2 . \end{aligned}$$

is the contribution to the energy from the long-range Coulomb-type field produced by the Bose-condensate.

It is easy to see how this contribution stabilizes the BEC at the critical point. Without this contribution the system would collapse due to the attractive self-interaction term, which for small $L$ scales as $\sim \hbar L_P^2 N^2/L^3$ and dominates over the first term in (34). We therefore ignore the latter term in stability analysis. Now, the same self-interaction energy localized within the region $L$ produces a Coulomb-type field that contributes into the energy through $H'$. This contribution scales as $E' \, \sim \, \hbar L_p^6 N^4 /L^7$. Correspondingly the $L$-dependence of energy is

36 $$\begin{aligned} E \, \sim \, \hbar L_p^6 N^4 /L^7 \, - \, \hbar L_P^2 N^2/L^3, \end{aligned}$$

which stabilizes the system at $L \, = \, \sqrt{N} L_P$.

### Black hole formation as quantum phase transition

The picture of a black hole as of BEC makes it clear why any scattering process that results into a black hole formation implies *classicalization* of gravity [24–26]. From what we said above, it follows that any such process can be understood as the formation of a graviton BEC and its evolution to the critical point of quantum phase transition.

In order to fix ideas let us consider a two-particle $|in \rangle$-state characterized by a center of mass energy $E$ and some impact parameter $b$. The gravitational self-energy of this system is $E_{gr}\, = \, \frac{E r_g(E)}{b}$, where by $r_g(E)$ we mean the gravitational radius corresponding to energy $E$ i.e. $r_g(E)\, = \, E L_P^2$. Let us consider the initial situation with very high total energy $E\, \gg \, \hbar / L_P$ and large impact parameter $b>>r_g(E)$. Under these conditions the gravitational self-energy $E_{gr}$ is much smaller that the total energy $E$. Irrespectively of this we can describe the gravitational self-energy in terms of a gas of $N$ gravitons with occupation number $N\, =\, \frac{Er_g}{\hbar }$ and typical wavelength $L \, = \, b$. We can consider this gas of soft gravitons as a BEC confined in a region of size $b$. What effectively plays the role of the confining potential for these gravitons is the external source, namely the two particles in the $|in \rangle$-state. Obviously, for such a condensate $\alpha N \, \ll \, 1$. Thus, we can assume that this BEC is under *weak coupling conditions* in a homogeneous phase. The classical order parameter solving the corresponding Gross–Pitaevskii equation effectively describes the Newtonian weak interaction among the $|in \rangle$-particles. In standard practice this corresponds to the eikonal approximation^3^. We wish to note that the BEC accounts for the exchange of $N$ gravitons in a ladder.

When we vary $b$, keeping the center of mass energy fixed, what we are doing is changing the coupling $\alpha$ among the gravitons in the BEC produced by the center of mass energy. To account for this increase in the coupling, using diagrammatic terms, we need to add graviton exchanges among two consecutive rungs of the eikonal ladder. According to our previous discussion we should expect to reach a critical value at which the condensate of gravitons approaches a point of quantum phase transition and becomes self-sustained. This obviously happens when $E$ and $E_{gr}$ are of the same order, or equivalently when $\alpha \, \sim 1/N$. At this point the system is fully dominated by self-gravity and *classicalizes*.

Again the diagramatic interpretation of this quantum phase transition is quite natural, namely the appearance of a contribution to the imaginary part of the amplitude. The black hole works as a bound state contributing to the imaginary part of the scattering amplitude. The special feature of the quantum phase transition is that the black hole *‘eats up’* the Goldstone mode in order to gain entropy. In other words the quantum phase transition of the gravitational BEC unitarizes the ultra-planckian scattering.

## Maximal packing: Bekenstein and Hawking

In this section we would like to clarify why the quantum holographic degrees of freedom that we were able to identify in our quantum picture would be impossible to recover in any semi-classical treatment. The two major pillars (as well as mysteries) of black hole physics are Bekenstein entropy and Hawking radiation.

Bekenstein tells us that the black hole entropy must scale as the area $S \, \propto \, L^2$. But, since entropy is dimensionless, the area must be measured in some units. In pure (quantum) gravity the only fundamental parameter of correct dimensionality is the Planck area, $L_P^2$. So the entropy must scale as $S \, \propto \, L^2/L_P^2$. But $L_P$ is a *quantum* length. So Bekenstein entropy is an intrinsically quantum entity—not semi-classical, but quantum. In particular, in both the classical and the semi-classical limits, which are commonly applied to black hole physics, the Bekenstein entropy becomes infinite. This is because in both limits, $L_P \, \rightarrow \, 0$. One may find this puzzling, but there is nothing to be scared of. This is exactly how it should be. In fact, this behavior is one of the consistency checks of Bekenstein’s entropy formula (see below). Notice that one cannot assume that this infinity will be regulated by some cutoff. This is because in gravity the cutoff length is $L_P$ and one should be able to consistently take it to zero.

In order to understand what is going on, let us focus on the semi-classical black hole limit. This limit is given by

37 $$\begin{aligned} G_N \, \rightarrow \, 0, ~~~ \, L \equiv M G_N \, = \, \mathrm{fixed} , ~~~ \hbar \, = \, \mathrm{fixed} , \end{aligned}$$

where $M$ is the black hole mass. In this limit the black hole geometry is fixed and one can consider quantum fluctuations on it without worrying about the backreaction. This is exactly the limit in which Hawking is doing his computation getting an *exactly* thermal spectrum of finite temperature $T \, = \, \hbar /R$. But notice that exactly in the same limit the Bekenstein entropy diverges, since $L_P$ is zero.

In order to explain why this situation is highly non-trivial and why its clarification requires the physics of BEC, we have to put ourselves in the place of a quantum observer. This observer sees an object of a finite size $L$ radiating a thermal spectrum, but having an infinite entropy, or equivalently, an infinite degeneracy of micro-states. If we think of these micro-states being formed by some quantum excitations about the black hole vacuum, we have to admit that each of this infinite number of distinguishable excitations should cost zero energy. How can an excitation localized within a finite size box cost no energy? Standard quantum mechanical intuition suggest that quantum excitation within the box of size $L$ should cost energy $\sim \, \hbar /L$. It is true that a finite size box can possess some zero modes, such as for instance Goldstone zero modes of broken translational invariance, but usually only a finite number of such modes exist. Thus, where are these infinite number of required zero modes coming from?

Our quantum picture answers this question in very simple physical terms. The finite size box can house an unlimited number of gapless modes, because it is a BEC with large occupation number $N$ and is at the critical point (3), or equivalently, at the point of maximal packing (5). As we have seen, thanks to this criticality, the collective Bogoliubov modes cost an energy given by (24), which is $1/\sqrt{N}$-suppressed relative to a naive expectation, $\hbar /L$. This immediately explains the infinite entropy of the box in the limit $N \, = \, \infty$.

With the above knowledge everything fits into its place. Hawking’s semi-classical limit (37) in our language is the double-scaling limit (8). In this limit the Bogoliubov energy gap collapses to zero and degeneracy becomes infinite, whereas the Hawking radiation becomes thermal. Correspondingly, the entropy of the black hole becomes infinite. Obviously, working in the semi-classical picture it is fundamentally impossible to trace the origin of holographic Bogoliubov degrees of freedom, since in this limit they decouple as $1/N$, as they should. In other words, any scattering experiment that is aiming to resolve these Bogoliubov modes must have an amplitude suppressed by powers of $1/N$.

In particular, Hawking’s famous ‘information paradox’ is an artifact of the semi-classical limit. For $N \, = \, \infty$ Bogoliubov modes can store an infinite amount of information, but it is also infinitely hard to retrieve this information, since modes are decoupled from any observer. But this is no more surprising than the fact that it is infinitely hard to find a needle in an infinite haystack.

## Quantum foundation of the holography

In our previous paper [1–3] we have suggested that the underlying quantum reason for holography was the equivalence of the system to large-$N$ BEC. These left open the question about the true quantum identity of the holographic degrees of freedom. Now we are in a position to suggest a very general answer to this question. We have seen that the graviton condensate that describes a black hole is at the critical point of a quantum phase transition. The quantum holographic degrees of freedom are degenerate Bogoliubov modes that become almost gapless at the critical point. It is striking that the physics at the critical point is described by some sort of CFT. The way black holes manage to store information with a minimal energy cost is through the Bogoliubov quasi-zero modes. The holographic bound becomes in this sense a bound on the available number of Bogoliubov zero modes for self-sustained condensates.

With the above observations, it is natural to generalize this connection beyond black holes to other gravitational or non-gravitational systems that are expected to have holographic description and in our language can be viewed as large-$N$ BEC. We then suggest that such systems are at the critical point of a quantum phase transition with holographic degrees of freedom being Bogoliubov modes. The first indication that we are on the right track comes in fact from generalizing our reasoning to AdS space. As we have shown, when viewed as a graviton condensate the AdS space also represents a critical point (maximal packing), with the same relation between the occupation number $N$, graviton coupling $\alpha$, and the length (radius of AdS) $L$ as in the black hole case. Remarkably, the occupation number of gravitons coincides with the value of central charge of CFT that has been conjectured [15–18] in AdS/CFT correspondence. Given the fact that the latter conjecture makes no appeal to the BEC nature of AdS space, the appearance of the same central charge in our approach is striking.

Our present picture suggests that this coincidence can have a deep underlying reason. The AdS space is a graviton BEC at the point of the critical phase transition. The appearance of a CFT description in such a system is very natural. This is the physics of the corresponding Bogoliubov modes.

Generalizing the above connection, we suggest the following quantum explanation of holography. A holographic description can naturally arise in non-perturbative field-theoretic systems, usually described by classical field configurations, which quantum-mechanically represent large-$N$ BECs at the critical point of quantum phase transition. The holographic description of such systems is in terms of a CFT-type theory of nearly gapless Bogoliubov modes. Notice that this holographic CFT in the $N \, \rightarrow \, \infty$ limit must become lower dimensional. This is because the appearance of the nearly gapless Bogoliubov modes is due to the transition from the uniform condensate to a phase of a bright soliton and is associated with the spontaneous breaking of the translational invariance. It is natural to expect that gapless Bogoliubov modes must be localized at the edge of forming a bright soliton where the gradients of the order parameter become maximal in the solitonic phase.

Finally, the quantum depletion of BEC should be a measure for the departure from the exact CFT. For example, the non-extremal black holes deplete and this affects the CFT description by $1/N$-effects. In contrast, the AdS and the extremal black holes can be protected from depletion by supersymmetry and extremality and this is probably the reason for a cleaner CFT description for such systems. This also suggests why the CFT description cannot work for de Sitter space.

## Outlook

The purpose of this work was to reformulate a quantum theory of black holes [1–3] in the language of condensed matter physics. The key point of the theory is to identify the black hole with a BEC of gravitons at the point of *maximal packing*. This term refers to a situation when the interaction strength $\alpha$ between the condensed Bosons (gravitons) is equal to the inverse occupation number $N$. It was suggested in a previous work that this property of maximal packing provides a quantum foundation to the known semi-classical properties of black holes. In particular, the quantum holographic degrees of freedom (flavors), responsible for the Bekenstein entropy and information storage, appear as collective nearly gapless excitations of the condensate.

In the present paper we have shown that the translation of the above picture in the more familiar language of condensed matter systems reveals that the physics of the maximally packed graviton condensate is the physics of BEC at the critical point of a quantum phase transition, very similar to what has been observed in cold atoms [10]. The quantum holographic degrees of freedom are nearly degenerate Bogoliubov degrees of freedom with a mass gap that scales $\sim \, 1/N$ instead of the inverse size of the system, as one would naively expect. The magic of the large-$N$ collective effect at the critical point allows one to have an unlimited number of nearly degenerate states within an arbitrarily small mass gap even for a fixed finite size of the system!

We have shown that the black hole graviton BEC remains at the critical point even during the quantum depletion and the collapse. This quantum collapse of the condensate is nothing but a quantum pre-cursor of the Hawking radiation.

It is important to realize that the above holographic degrees of freedom are not reducible to known semi-classical excitations in the background black hole metric. These are Bogoliubov modes of the graviton condensate itself, which are intrinsically quantum and must decouple as $1/N$ in the semi-classical limit (8).

Our results have a number of interesting implications. First, they point to a deep underlying connection between the maximally packed gravitational (or non-gravitational) systems and BECs at the critical point of the quantum phase transition.

This connection offers an intriguing possibility of simulating black hole physics in table-top experiments.

We have also pointed out that our findings suggest a very general quantum foundation of holography. According to this idea, non-perturbative field configurations that usually are treated classically in reality represent large-$N$ BEC’s at the critical point of a quantum phase transition. These systems admit a holographic description in the form of (exact or approximate) CFT of the gapless Bogoliubov degrees of freedom. Moreover, the departure from exact CFT must be measured by the quantum depletion properties of the condensate.

It would be interesting to apply this concept to different systems. The obvious choices of gravitational systems would be AdS and de Sitter spaces, which as we have shown in [1–3] obey the large-$N$ BEC properties similar to black holes.

However, we have also shown that the ordinary field theoretic topological defects, such as a ’t Hooft–Polyakov monopole, when viewed as a BEC also obey the condition of the maximal packing (5). Thus, these systems must also be equivalent to BECs at the critical point of a quantum phase transition. The corresponding Bogoliubov modes should then give a holographic description of such non-perturbative objects. This may shed new useful light on the physics of such objects.

## Footnotes

1 In the previous paragraph we have simply defined the black hole entropy as measuring the degeneration of the corresponding BEC ground state at the critical point. In addition to this microcanonical notion of entropy we can naturally define a quantum contribution. Denoting by $$|BEC(i)>$$ with $$i=1,\ldots ,N$$ the quasi-degenerate ground states at the critical point, we should think of the black hole quantum state as some quantum superposition $$|BH>= \sum _ic_i|BEC(i)>$$ with $$\sum _i|c_i|^2=1$$ and the corresponding density matrix as $$\rho =|BH>
<BH|$$. If we wash out the off-diagonal pieces of $$\rho $$ as we do when we measure the system we gain an amount of Von Neumann entropy of the order $$-\sum _i (c_i^2lnc_i^2) \sim lnN$$. This is the quantum contribution to the black hole entropy in the BEC portrait.

2 It is customary to try to describe black hole physics in terms of two complementary pictures [23]: the in-falling and the external observers. In a nutshell the two descriptions, within our scheme, are roughly as follows. The external observer sees the cascade of quanta of increasing energy that escape and the accompanying change of $N$. Instead, the observer inside the BEC experiences how the number of low-lying Bogoliubov modes diminishes but also how the $b$-operators defining the creation of these low-lying excitations are self similarly transformed along the process. The situation could become paradoxical if we insist in keeping the same operators for the Bogoliubov modes along the process i.e. if we do not track the self-similar change of the underlying Bogoliubov transform we have defined in the previous section.

3 Our aim here is not to enter into the technical subtleties of the trans-Planckian scattering, on which a lot of work has been done since the pioneering papers [27–30]. Our aim is to uncover the BEC picture of black hole formation in this scattering, which is the key to understanding classicalization of UV-gravity [24–26].
